# Patient Educational Materials for Pheochromocytoma Exceed Recommended Readability Level: An Analysis Across Three Independent Reading Instruments

**DOI:** 10.1007/s13187-025-02666-3

**Published:** 2025-06-21

**Authors:** Valerie L. Armstrong, Kelsey L. Grabeel, R. Eric Heidel, Christel M. McGillicuddy, Polina Zmijewski, Peyton Murdock, John I. Lew, Tanaz M. Vaghaiwalla

**Affiliations:** 1 Department of Surgery, Mayo Clinic Arizona, Phoenix, AZ USA; 2 University of Tennessee Graduate School of Medicine, University of Tennessee Medical Center, Knoxville, TN USA; 3 DeWitt Daughtry Family Department of Surgery, Division of Endocrine Surgery, University of Miami Leonard M. Miller School of Medicine, 1120 NW 14th Street, CRB 4th floor (M-875), Miami, FL 33136 USA

**Keywords:** Readability, Health literacy, Patient education, Adrenal tumor, Neuroendocrine tumor

## Abstract

Patient education is a critical component of effective medical care; > 50% of patients seek medical information from online sources. The recommended reading level is sixth grade. This study examines reading level of current online educational resources for patients with pheochromocytoma. A review of 50 online patient educational materials related to pheochromocytomas was performed. Using three validated instruments: Flesch Reading Ease (FRE), Simplified Measure of Gobbledygook (SMOG), and Gunning Fog Index (GFI) scores, the reading grade level was calculated, and Spearman correlations were performed to evaluate associations between the reading instruments. Thirty-two online patient educational materials were included. The majority of the materials were from academic and research institutions (47%), followed by general information and overviews (19%), government health resources (9%), cancer-specific organizations (9%), support and advocacy groups (9%), and children’s health (6%). There were no patient materials that were written at the appropriate grade level. Materials were above a twelfth-grade reading level in 61% of sources (FRE = 78.2%, SMOG = 40.6%, GFI = 59.4%). There were strong, positive correlations between the three reading instruments utilized in the analysis. Readable educational materials facilitate effective healthcare literacy and empower patients to participate in health care decisions, which is especially important given the complex diagnostic and therapeutic considerations for pheochromocytomas. This study is the first to assess readability of online educational resources for patients with pheochromocytoma and reveals a significant gap in the availability of materials that meet the recommended grade level. Developing online education materials that meet the recommended reading grade level is essential to inform patient education and shared decision-making.

## Introduction

According to the National Literacy Institute, an estimated 21% of adults in the USA are illiterate, and more than 50% currently have a literacy level below a sixth-grade level in 2024. The National Work Group on Cancer and Health, the American Medical Association, and the National Institutes of Health recommend a reading level at the sixth-grade for patient educational materials [1, 2]. The internet is a frequently used source of health care information. Data from the National Center for Health Statistics estimate that 58.5% of adults in the USA use the internet to seek health information to better understand their diagnosis and management options [3]. Although there has been an increase in the volume, access, and programs to search for patient educational materials, their utility may be limited by their readability for patients [3]. Previous studies have demonstrated the scarcity of online patient educational material at or below the recommended sixth-grade reading level across numerous surgical fields, including otolaryngology [4, 5], orthopedic surgery [6], bariatric surgery [7], hand surgery [8], and ophthalmology [9].

For patients with rare or complex surgical conditions, there may be a greater need for patient education materials that meet the recommended readability level. Pheochromocytomas, which are tumors that arise from the adrenal medulla, are complex malignancies with an incidence of only 0.8 per 100,000 person-years [10]. Reports estimate that one in 3,000 patients will be diagnosed with a pheochromocytoma or paraganglioma [11]. The diagnosis is made based upon biochemical workup with confirmation of catecholamine hypersecretion and imaging studies to identify the tumor location [12]. Patients require multidisciplinary care for medical optimization prior to surgical resection, specialized operative teams for management of hemodynamics during surgery, and long-term follow up for surveillance [13, 14]. Patients with complex conditions like pheochromocytomas, which often represent a diagnostic challenge and require multimodal treatment, may have a greater need for comprehensible educational materials to facilitate constructive discussions with their clinicians [15].

Studies show that patients desire understanding of their illnesses [12] and will often obtain information from readily available online sources [16]. In order to provide patient-centered health care, an understanding of their disease is necessary [2, 12]. Therefore, the development of educational materials at the recommended reading level is a critical component to inform shared decision-making between patients with a diagnosis of pheochromocytoma and their treating physician. This study uses three independent reading instruments to examine the reading level of current online educational resources for patients with pheochromocytoma [3, 17–20].

## Materials and Methods

In order to identify the freely accessible online patient educational material on pheochromocytoma, a Google search was conducted in September 2024 by three individuals who independently reviewed each of the sources. To remove targeted results or the influence of previous searches, public computers were used, and browsing history was cleared. The phrases “pheochromocytoma,” “adrenal pheochromocytoma,” and “I have a pheochromocytoma” were used to conduct the search.

The first 50 unique sources were recorded and later assessed for inclusion in this project. Sources were included if they contained information on workup, treatment options, risks of surgery, and outcomes. Sources were excluded if they required payment or subscription to access, open forums or discussions, personal pieces from other patients or celebrities, commentary, videos, news pieces, or were written primarily in any language other than English. Sources were categorized based on the institution or website from which they originated. The categories included government and institutional, health resources, academic and research institutions, cancer-specific organizations, support and advocacy groups, general information and overviews, and children’s health.

Three validated instruments to assess the readability of each website were used: the Flesch Reading Ease (FRE) Score, the Simplified Measure of Gobbledygook (SMOG) Score, and the Gunning Fog Index (GFI). All three scores were calculated by a medical librarian (Table 1).

**Table 1:**
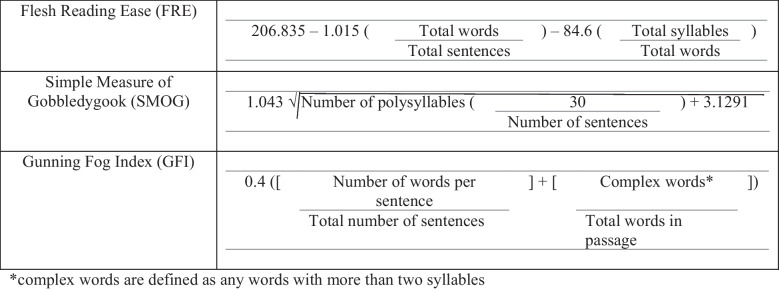
Readability formulas

The *FRE Score* was developed in 1948 and is used by the Department of Defense. It involves an evaluation of readability by calculating ratios of total words to total sentences and syllables to the overall word count. Higher scores indicate material that is easier to read [17, 21].

The *SMOG Score* was used to assess reading grade level. This is done by selecting 10 sentences from the beginning, middle, and end of the text to assess a total of 30 sentences. Within the 30 sentences, a tally of all words with three syllables or longer is recorded. The square root of the total tally is conducted, and three is added to this number, which is the reading grade a reader must have obtained to understand the text [18]. The SMOG formula is well suited for this study because of its consistent results and validation criteria for determining reading grade level estimates [19].

The *GFI* was created in 1952 by Robert Gunning and is cited to have an 80% accuracy in predicting the difficulty of a written passage. The formula used is similar to other readability scores in that it assesses the length of sentences in a passage as well as the percentage of polysyllabic words. The result of this formula is the grade a reader would have to obtain in order to understand the written text [20].

The prevalence of appropriate reading grade levels was calculated using frequency and percentage statistics. Means (M) and standard deviations (SD) were reported for each instrument score. Spearman correlations were performed to evaluate associations between reading instruments. Statistical significance was assumed at an alpha value of 0.05, and all analyses were performed using SPSS Version 29 (Armonk, NY: IBM Corp.).

## Results

**Fig. 1:**
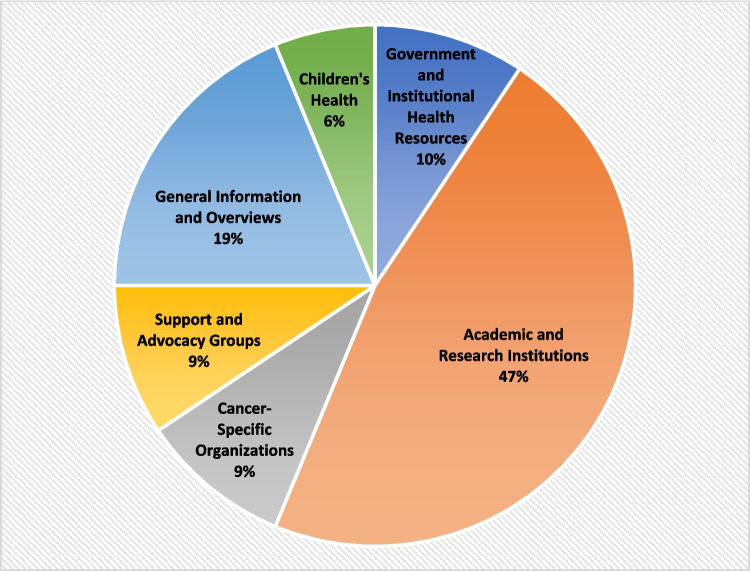
Sources of the patient education materials for pheochromocytoma

From the first 50 unique websites queried for pheochromocytoma diagnosis using the keywords “pheochromocytoma,” “adrenal pheochromocytoma,” and “I have a pheochromocytoma” used to conduct the search, a total of 32 sources were deemed to be patient education materials by the three independent evaluators and were included in the study analysis. A majority (47%) of the patient education materials were from academic and research institutions, followed by general information/overviews (19%), and the remainder from government health resources (9%), cancer-specific organizations (9%), support and advocacy groups (9%), and children’s health (6%) sources (Fig. 1).

**Fig. 2:**
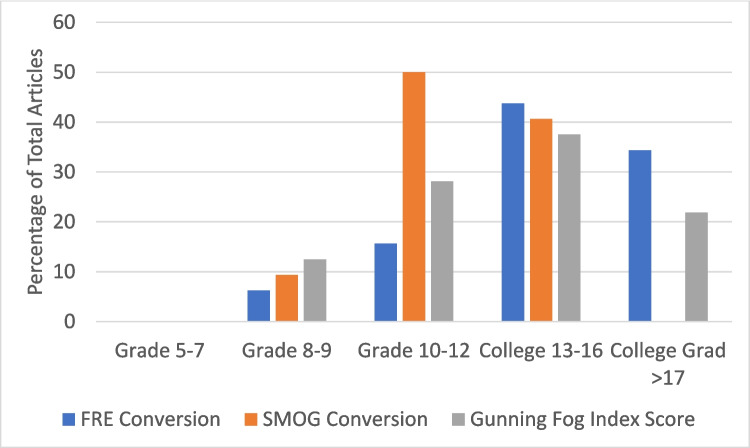
Percentage of reading levels across the patient education materials for pheochromocytoma Acronyms and descriptions: FRE, Flesch Reading Ease; GFI, Gunning Fog Index; SMOG, Simplified Measure of Gobbledygook

After calculating the numerical value of all three scores, a conversion score was obtained. A score of 1 correlated to reading grades five through seven, 2 correlated to grades eight and nine, 3 correlated to grades 10–12, 4 correlated to grades 13–16 (college level), and 5 was the highest score, which correlated to grade ≥ 17 (professional level). The mean FRE conversion was *M* = 4.06 (SD = 0.88), the mean SMOG conversion was *M* = 3.3 (SD = 0.64), and the mean GFI conversion was *M* = 3.7 (SD = 0.97) (Fig. 2).

A majority (61%) of the reading materials in the study were above a twelfth-grade reading level (FRE 78.2%, SMOG Conversion 40.6%, Gunning Fog Index 59.4%). There were no materials written at or below the recommended sixth-grade level; the lowest reading grade level was found for one educational source that was written at an eighth-grade level [21]. There were strong, positive correlations between the readability instruments: FRE and SMOG (*rs* = 0.78, *p* < 0.001), FRE and Gunning Fog (*rs* = 0.81, *p* < 0.001, and SMOG and Gunning Fog (*rs* = 0.73, *p* < 0.001), indicating their precision in determining the reading grade level of the materials. In addition to their precision, their accuracy has been previously discussed and verified across other studies [17–19, 21, 22].

## Discussion

Although the current recommended levels for readability are at or below a sixth-grade level [20], this study reveals that patient education materials fail to meet these standards and identifies a significant obstacle for patients with a diagnosis of pheochromocytoma who may be seeking comprehensible educational resources. To the authors’ knowledge, this study represents the first in the literature to examine the readability of online resources for patients with pheochromocytoma.

Despite extensive evidence demonstrating the need for more readable patient educational materials, limited progress has been made in the field of endocrine surgery. A recent study published on the readability of internet-based patient education materials related to parathyroid surgery demonstrated similar results; of 26 analyzed sources, no single source was at the recommended reading level [3]. Echoing these results, studies examining online patient education materials related to the management of thyroid nodules, surgery, and ablation failed to demonstrate available online materials at the appropriate reading grade level [23–26]. Finally, studies evaluating the readability of the educational resources related to adrenal disease remain even more scarce. Therefore, the authors’ study provides an important addition to the literature, demonstrating that current educational resources available to patients with a diagnosis of pheochromocytoma do not meet the recommended reading level.

Effective patient education has numerous benefits. It enables empowerment of individuals to participate in their own healthcare decisions, which is especially important for patients with pheochromocytoma given the complex diagnostic and therapeutic considerations patients may encounter during their care. As a result, there have been many proposals to improve upon the readability of patient education materials. First, some authors suggest utilizing artificial intelligence to revise the currently available online materials in order to decrease the laborious process of re-writing. One study demonstrated that a freely available online artificial intelligence dialog platform could successfully convert preexisting patient education materials to a more appropriate reading level [27]. In a study by Eisinger et al., large language models such as the artificial intelligence-generated GPT-4 demonstrated success in transforming patient educational materials to a more comprehensible format. In this study, the authors tested the utility in transforming hospital discharge letters into a more readable, patient-centered text  [28]. These findings were similarly echoed across other studies utilizing GPT-4’s ability to generate patient-centered letters based on discharge documents or brief typed-out clinical instructions [29, 30].

 The development of patient educational materials with rigorous evaluation to ensure the recommended reading grade level is widely recommended [12, 16, 24–26]. In addition to training healthcare providers  in assessing health literacy [31], strategies to improve readability of patient education materials may include avoiding long sentences containing more than 8 to 10 words [32], utilizing illustrations and font sizes between 12 and 14 points [33], and teach-back techniques for in-person patient encounters [34, 35]. Furthermore, Badarudeen et al. have suggested utilizing readability formulas in patient education materials prior to publishing them [36]. In a study by Cote et al. examining readability of materials on thyroid disease, the authors have suggested incorporating the discussion of patient materials during the visit with the patient to ensure both patient and physician are aware of what health care information is being accessed [2]. In addition to the extensive need for improvement in website content and design to improve the readability and utility of patient educational resources for pheochromocytoma, physician–patient education is critical in the patient’s understanding of their illness. Patients with rare diseases should not only have access to appropriate educational materials, but they should be referred to specific advocacy groups to promote a sense of community and to provide additional resources [24].

The authors’ study has several limitations. Articles such as those from scientific journals, blog posts, news, interviews, and commentary were excluded from analysis in this study. These sources, while not recommended to be the primary source of education for patients, may be utilized by patients to a degree that is underrecognized. Additionally, there may be limitations arising from the readability formulas used to score the articles in this study. While all of the formulas used have been validated and are frequently used to study the readability and complexity of written text across multiple different fields, the authors’ study may not fully capture the complexity of medical information. These formulas may not account for factors such as sentence structure, presence of medical jargon that may affect readability, or other factors including layout, graphics, and charts. The authors’ sought to mitigate these limitations by using three independent reading instruments in this analysis. Nevertheless, this study represents the first in the literature to evaluate education materials for patients with pheochromocytoma, and the authors’ study highlights a critical gap in the current materials available for patients.

In conclusion, online websites are a commonly used source of health information for the general population. This analysis reveals that the readability of current education materials for patients with pheochromocytoma is consistently higher than the recommended reading grade level. The use of readability instruments may be of benefit to authors when preparing patient education materials. Efforts are needed to develop patient education materials that adhere to the recommended readability level. Developing readable materials for patients with pheochromocytoma is crucial to clinicians in order to improve healthcare access and quality, as well as facilitate shared decision-making and surgical planning.
